# Epigenetic silencing of ZIC4 unveils a potential tumor suppressor role in pediatric choroid plexus carcinoma

**DOI:** 10.1038/s41598-024-71188-7

**Published:** 2024-09-12

**Authors:** Dina Hesham, Amal Mosaab, Nada Amer, Nouran Al-Shehaby, Sameh Magdeldin, Ahmed Hassan, Hristo Georgiev, Hisham Elshoky, Mona Rady, Khaled Abou Aisha, Ola Sabet, Shahenda El-Naggar

**Affiliations:** 1 Tumor Biology Research Program, Basic Research Unit, Research Department, Children’s Cancer Hospital in Egypt 57357, 1 Sekket El Emam, El Madbah El Kadeem Yard, Sayeda Zeinab, Cairo, Egypt; 2 Microbiology, Immunology and Biotechnology Department, Faculty of Pharmacy and Biotechnology, German University in Cairo (GUC), Cairo, Egypt; 3 Proteomics and Metabolomics Research Program, Basic Research Unit, Research Department, Children’s Cancer Hospital Egypt 57357, Cairo, Egypt; 4 Department of Physiology, Faculty of Veterinary Medicine, Suez Canal University, Ismailia, Egypt; 5 Institute of Immunology, Hannover Medical School, Hannover, Germany; 6 Faculty of Biotechnology, German International University, New Administrative Capital, Cairo, Egypt; 7 Division of Immunology, University Children’s Hospital Zurich, Zurich, Switzerland

**Keywords:** ZIC4, Pediatric choroid plexus carcinoma, Epigenetic silencing, Tumor suppressor, Transcriptomic profiling, Proteomic profiling, Cancer, Cell biology, Molecular biology

## Abstract

Zic family member *ZIC4* is a transcription factor that has been shown to be silenced in several cancers. However, understanding the regulation and function of *ZIC4* in pediatric choroid plexus tumors (CPTs) remained limited. This study employed data mining and bioinformatics analysis to investigate the DNA methylation status of *ZIC4* in CPTs and its correlation with patient survival. Our results unveiled *ZIC4* methylation as a segregating factor, dividing CPT cohorts into two clusters, with hyper-methylation linked to adverse prognosis. Hyper-methylation of *ZIC4* was confirmed in a choroid plexus carcinoma-derived cell line (CCHE-45) by bisulfite sequencing. Furthermore, our study demonstrated that demethylating agent and a histone methyltransferase inhibitor could reverse *ZIC4* silencing. RNA sequencing and proteomic analysis showed that *ZIC4* over-expression influenced genes and proteins involved in immune response, antigen processing and presentation, endoplasmic reticulum stress, and metabolism. Functionally, re-expressing *ZIC4* negatively impacted cell proliferation and migration. Ultimately, these findings underscore *ZIC4* hyper-methylation as a prognostic marker in CPTs and shed light on potential mechanisms underlying its tumor suppressor role in CPC. This insight paves the way for novel therapeutic targets in treating aggressive CPTs.

## Introduction

Pediatric choroid plexus tumors (CPTs) are rare intraventricular central nervous system (CNS) neoplasms originating from the choroid plexus epithelium. They account for less than 1% of all intracranial tumors and 2–4% of brain tumors in children^1^. Typically, CPTs affect patients under three years of age, with 10 to 20% diagnosed within the first year of life^2^. According to the World Health Organization (WHO) classification, CPTs are classified into three grades: benign choroid plexus papilloma (CPP) (WHO grade I), the intermediate form atypical choroid plexus papilloma (ACPP) (WHO grade II), and the aggressive choroid plexus carcinoma (CPC) (WHO grade III)^3^. Current evidence supports the involvement of TP53, Notch signaling, and Sonic Hedgehog (Shh) in the pathogenesis of CPTs. However, the exact molecular mechanisms underlying CPT development remain largely uncharacterized^4^.

Pediatric cancers can be considered as a consequence of disruptions to normal development. During embryonic development, many biological processes and cellular differentiation depend on epigenetic regulation^5,6^. Over the recent decades, an increasing amount of evidence supports the role of epigenetics in tumorigenesis, raising the possibility that understanding these epigenetic mechanisms may contribute to classification, risk stratification, and novel therapies^7,8^. Epigenetic silencing of tumor suppressors has been reported to induce carcinogenesis-related molecular alterations, including abnormal cell division, genomic instability, cellular immortality, metabolic reprogramming, metastasis, and tumor plasticity^9^.

Recently, a methylation profiling study was conducted on tissue samples from CPTs, categorizing different pathological subtypes into two clusters: group A (CPP and ACPP) and group B (all CPC and some CPP and ACPP)^10^. This approach facilitated the segregation of typically challenging atypical tumors (ACPPs) into categories with either favorable outcomes (group A) or poor outcomes (group B)^11,12^. Notably, the most significant differentially methylated region (DMR) between groups A and B encompassed the *ZIC4* gene. Furthermore, hyper-methylated positions were identified in both the promoter region and the gene body.

The *ZIC* gene family, comprising *ZIC1, ZIC2, ZIC3, ZIC4,* and *ZIC5*, encodes multifunctional transcriptional regulators required for embryogenesis and plays roles in regulating morphogenesis, cell proliferation, and cell homeostasis^13–15^.

In the last decade, the role of *ZIC* genes has been increasingly recognized in several cancers^16–22^. *ZIC4* has been described to be epigenetically silenced in many types of cancers which was correlated with poor prognosis, and was shown to have a tumor suppressor function by being involved in pathways like Shh and notch pathways^23–26^. Recently, epigenetic silencing of *ZIC4* was demonstrated to involve both DNA methylation and histone modification which was found to be crucial in the development and progression of hepatocellular carcinoma (HCC), and its over-expression reduced proliferation and invasiveness of HCC cells^27^.

In this study, we aim to explore whether *ZIC4* exhibits the same epigenetic regulation in CPTs and to understand the mechanistic impact and underpinnings of *ZIC4* methylation status using a CPC-derived in vitro model system. *ZIC4* methylation status segregated CPT cohorts into two clusters, with hyper-methylation linked to inferior prognosis. We further validated that *ZIC4* is epigenetically silenced in our CPC-derived cell line (CCHE-45). Moreover, by re-expressing ZIC4 in CPC cells, we could identify a set of key molecular targets involved in its tumor suppressor function in the context of CPTs. ZIC4 exerts tumor suppressor activity in CCHE-45 by up-regulating genes involved in immune response modulation pathways such as RIGI-like receptor signaling and IFN signaling, as well as influencing genes and proteins associated with oxidative stress and metabolic pathways.

## Results

### *ZIC4* methylation status segregates pediatric CPTs into two subgroups and correlates to survival outcomes

**Fig. 1:**
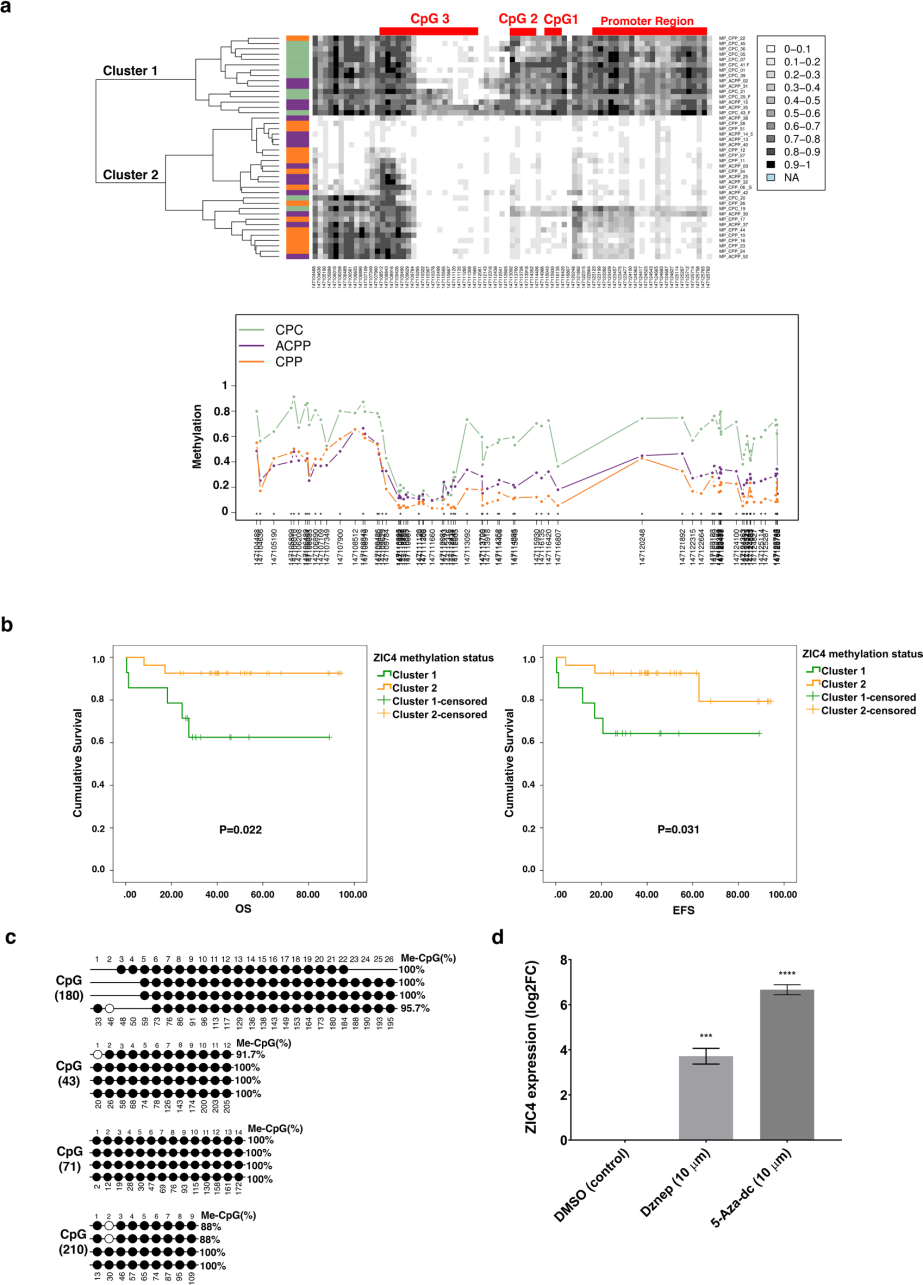
ZIC4 methylation status among choroid plexus tumors (CPTs) and CCHE-45 cell line. (**a**) β-values of *ZIC4* methylation probes segregate CPTs into 2 clusters: high-methylation "cluster 1" and low-methylation "cluster 2", as shown in the dendrogram of the GSE156090 cohort. The positions representing CpG islands and promoter regions are highlighted in red. In the methylation plot, each line represents the methylation mean for each position for each group of samples. Asterisks indicate a statistical significance as calculated by the Kruskal–Wallis test. The coloring of the samples was according to the pathology (CPC = green, ACPP = purple, and CPP = orange). (**b**) In the GSE156090 cohort, patients in the *ZIC4* high-methylation group (cluster 1) had shorter overall and event-free survival compared to the *ZIC4* low-methylation group (cluster 2) by Kaplan–Meier. (**c**) Bisulfite sequencing analysis of *ZIC4* CpG islands in CCHE-45. The open and filled circles indicate the unmethylated and methylated CpGs, respectively (no. of colonies sequenced per island = 4). (**d**) *ZIC4* mRNA expression in CCHE-45 treated with 10 μM 5-aza-dC (n = 3) and 10 μM Dznep (n = 3) versus DMSO.***P < 0.001, ****P < 0.0001.

Initially, we thought to mine the data already available in the genome-wide methylation data of two independently published pediatric CPT studies: GSE61044 (n = 30) and GSE156090 (n = 42). In GSE61044, the study was carried out using a 450 K methylation array that encompassed 64 probes for *ZIC4*, while in GSE156090, they utilized an EPIC 850 K methylation array where *ZIC4* was represented by 77 probes (Table S1). The probes covered promoter, CpG islands, coding, and non-coding regions. The analysis revealed that the *ZIC4* methylation pattern could segregate pediatric CPTs into two subgroups: cluster 1 and cluster 2. In both CPT cohorts, cluster 1 had a higher methylation pattern than cluster 2, especially at the promoter region and CpG islands 1 and 2 (Fig. 1a, Fig. S1). Notably, cluster 1 included mainly CPC cases, some ACPP -in the Amer et al. cohort (GSE156090)- and a few CPP. Kaplan–Meier analysis of the GSE156090 cohort showed that cluster 1 had significantly shorter overall and event-free survival rates (*p* value  = 0.022 and *p* value = 0.031, respectively) than cluster 2 (Fig. 1b). Altogether, these results suggested that *ZIC4* hypermethylation is correlated with a poor prognosis. To confirm that *ZIC4* was epigenetically silenced in a CPC-derived cell line, the expression of ZIC4 was examined in CCHE-45 cells (Fig. S2). To validate that the absence of *ZIC4* expression was due to methylation, we performed bisulfite sequencing of all corresponding CpG islands (Fig. 1c, Fig. S3a, Table S2). Moreover, *ZIC4* expression was rescued after treating CCHE-45 with a demethylating agent 5-Aza-dc (Fig. 1d). Taken together, these results confirmed that the absence of ZIC4 in CCHE-45 is due to epigenetic silencing by hypermethylation. EZH2-dependent histone methylation was previously reported to contribute to *ZIC4* repression^27^. Computationally, the EZH2 binding site on *ZIC4* was detected at the promoter region (Chr3:147,123,922–147,123,931) as inferred by MotEvo (Fig. S3b). To examine if this mode of epigenetic regulation also affects *ZIC4* expression, CCHE-45 cells were treated with Dznep, an inhibitor of EZH2. Immunoblotting for EZH2 and H3K27me3 was conducted to demonstrate the efficiency of Dznep to inhibit both the activity and expression of EZH2, as well as reduction in trimethylation levels of H3K27 after Dznep treatment. The results confirmed diminished levels of EZH2 and a decrease in H3K27me3, validating the functional impact of Dznep on histone methylation (Fig. S3c). Dznep restored *ZIC4* expression, albeit not to the same extent as when a direct demethylating agent was used (Fig. 1d).

### ZIC4 up-regulates the expression of genes involved in interferon signaling and antigen presentation

**Fig. 2:**
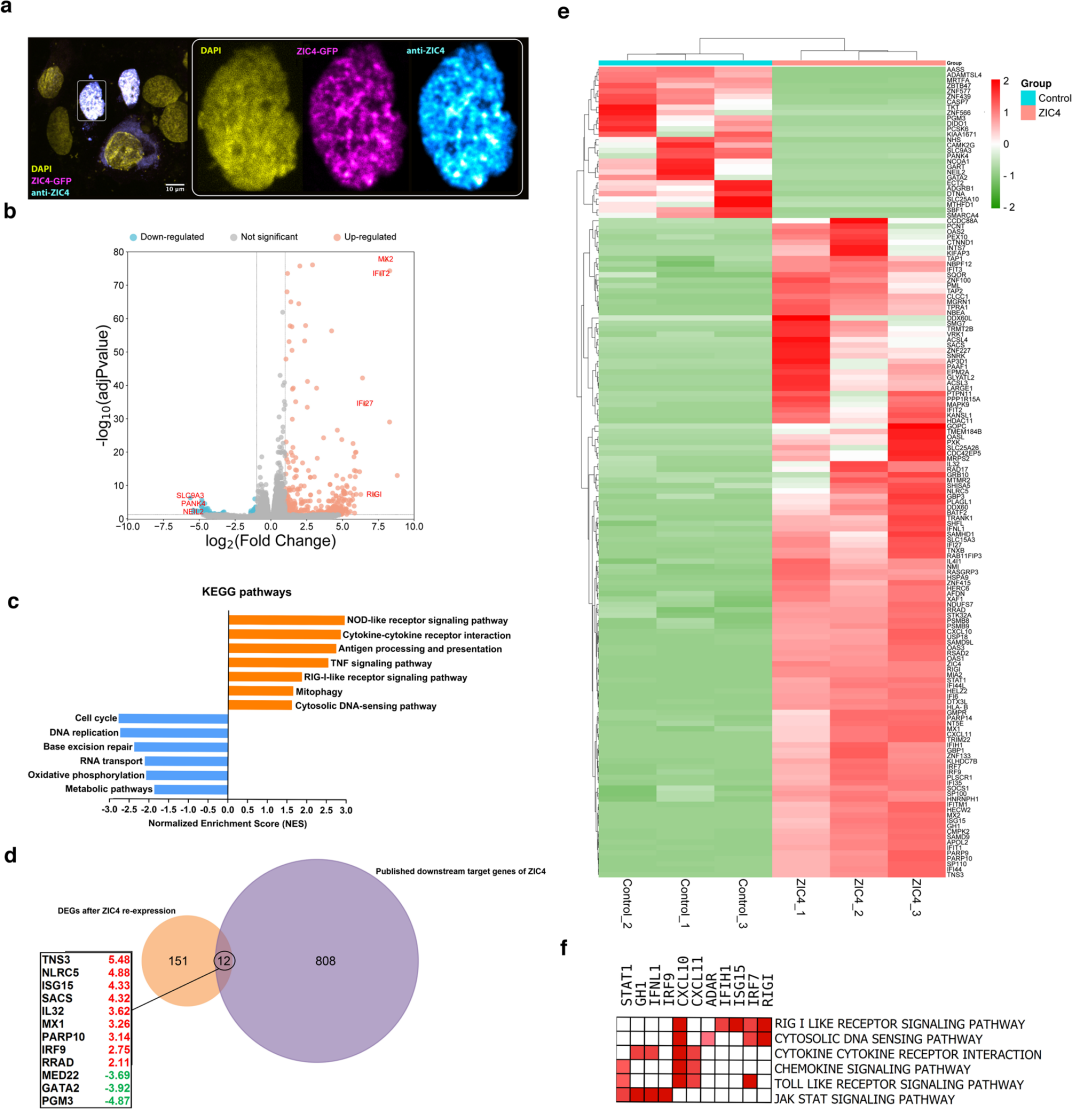
Transcriptomic profiling of CCHE-45 after over-expression of ZIC4. (**a**) Immunofluorescence imaging showing the absence of ZIC4 in untransfected cells, while ZIC4 is detected in the nucleus of the transfected cells, particularly in transcriptionally active regions. (DAPI staining in Yellow, GFP tag in Magenta, and ZIC4 in Cyan). (**b**) Volcano plot of genes after ZIC4 over-expression compared to the control. (**c**) Gene Set Enrichment Analysis (GSEA) of significant differentially expressed genes (DEGs) (adjusted *p* value ≤ 0.05). The figure shows the NES (normalized enrichment score) of the top relevant enriched KEGG pathways (blue bars) and top relevant depleted pathways (orange bars) in ZIC4-transfected versus control. (**d**) Venn diagram showing the common genes from the differential expression analysis after ZIC4 over-expression and published downstream targets of ZIC4. (**e**) Heatmap of top 150 DEGs (adjusted *p* value ≤ 0.05 and fold change between 1.5 and − 1.5). Red represents the higher expression, and green represents the lower expression of each gene in all six samples. The hierarchical clustering was performed using the average linkage method with Euclidean distance measures. (**f**) Leading edge analysis of KEGG pathways of up-regulated DEGs (red denotes up-regulation of DEGs ZIC4-transfected versus control).

Using a GFP-tagged ZIC4 vector, we transiently re-expressed ZIC4 in CCHE-45 cells and validated its expression using western blot (Fig. S4). Immunofluorescence imaging showed that ZIC4 was primarily localized to the nucleus, specifically the nucleoplasm, with a spatial pattern reminiscent of transcriptionally active euchromatin (Fig. 2a). On the functional side, ZIC4 over-expression significantly inhibited the proliferative capacity of CCHE-45 cells relative to mock control (p < 0.05), as assessed using the cell viability MTT assay (Fig. S5a). Other cancer traits include migratory potential, assessed using a wound-healing scratch assay. In the presence of ZIC4, wound closure was slower in CCHE-45 cells than in mock control cells, indicating reduced migratory potential (Fig. S5b). To investigate the biological impact of silencing *ZIC4* in CPTs, we first analyzed data from publicly available databases for its downstream targets (Table S2). These target genes were inferred computationally or verified experimentally (chromatin immunoprecipitation sequencing) from published studies^28^. Protein–protein interaction (PPI) analysis of these genes revealed a main PPI network (Fig. S6a). Further, these genes were found to be involved in various pathways, including metabolic pathways, calcium signaling pathways, ubiquitin-mediated proteolysis, apoptosis, spliceosome, as well as many cancer-related signaling pathways like hippo, HIF-1, PI3K-Akt, and Shh signaling pathways (Fig. S6b, Table S3). Next, to gain more insight into the function of ZIC4 in CPC, we performed RNA sequencing to compare mock control and ZIC4-transfected CCHE-45 cells. We identified 1818 differentially expressed genes (adjusted *p* value < 0.05) (Fig. 2b, Table S4). Gene set enrichment analysis (GSEA) revealed that KEGG pathways enriched after ZIC4 over-expression were mainly involved in immune response signaling, antigen processing, and presentation. On the other hand, depleted KEGG pathways included cell cycle, DNA replication, RNA transport, oxidative phosphorylation, and metabolic pathways (Fig. 2c, Table S4). Further filtering of the differentially expressed genes (DEGs) by fold change (between 1.5 and − 1.5), identified 163 genes (130 up-regulated, 33 down-regulated). When we compared our DEGs data with the published *ZIC4* downstream targets, 12 genes were found to be in common (Fig. 2d). Many of the top-up-regulated genes were related to interferon signaling, such as 2,5-oligoadenylate synthetase (*OAS*), myxovirus resistance gene (*MX*), interferon regulatory factor (*IRF*) family genes, XIAP associated factor 1(*XAF1*) and signal transducer and activator of transcription 1(*STAT1*). In addition, genes associated with antigen processing and presentation such transporter 1 and 2, ATP binding cassette subfamily B member (*TAP1* and *TAP2*), major histocompatibility complex class I, B (*HLA-B*) and immune-proteasome subunits (*PSMB8* and *PSMB9*) were among the up-regulated genes (Fig. 2e, Table S4). GSEA and leading-edge analysis showed that the top up-regulated genes were involved in RIG-I-like receptor signaling pathway, cytosolic DNA sensing pathway, cytokine-cytokine receptor interaction, chemokine signaling pathway, toll-like receptor signaling pathway, and JAK-STAT signaling pathway (Fig. 2f).

### ZIC4 alters the expression of proteins involved in response to ER stress, metabolism and splicing in CCHE-45

**Fig. 3:**
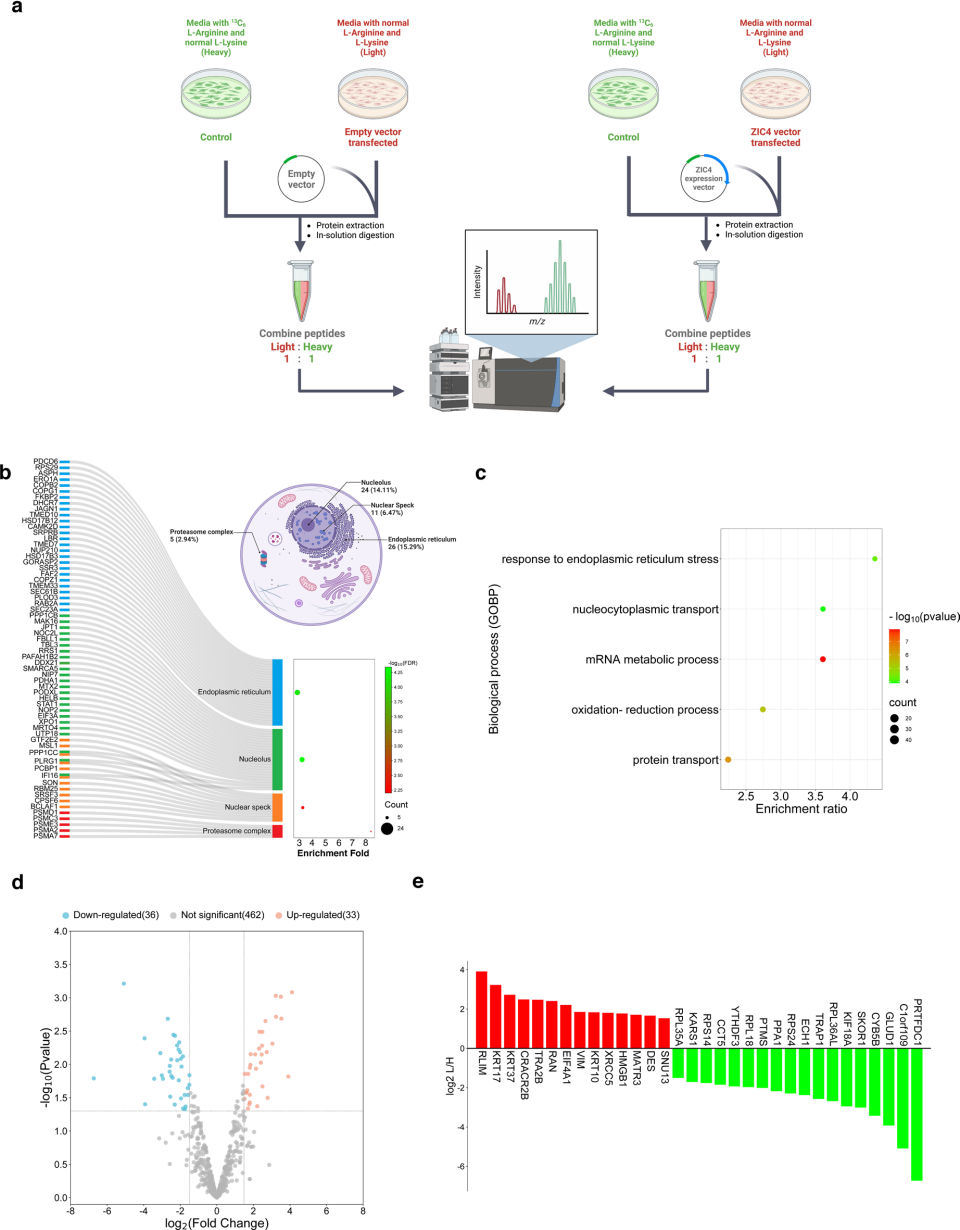
Proteomic analysis after over-expression of ZIC4 in CCHE-45. (**a**) Workflow of the quantitative SILAC proteomics performed for mock-transfected and ZIC4-transfected cells, compared to control cells. Control cells were grown in media supplemented with "heavy" arginine isotope, while treated cells were grown in media supplemented with "light" arginine. (**b**) Subcellular localization of proteins was detected only after ZIC4 over-expression. Percentage denoted the proportion of proteins in each compartment relative to the total number of proteins detected exclusively after ZIC4 over-expression (n = 170). Sankey diagram was used to visualize top enriched subcellular compartments and the proteins annotated within. In the bubble chart, the color and size of the points were scaled with respect to enrichment FDR and the count of related proteins. (**c**) Biological processes (GOBP) enriched by proteins detected only after ZIC4 over-expression in CCHE-45. In the bubble chart, the size of the points corresponds to the count of proteins, and the color of the points was scaled with respect to the enrichment *p* value. (**d**) Volcano plot of the up-regulated and down-regulated DEPs after ZIC4 over-expression compared to the control. In the plot, the x-axis represents the log2 fold change, and the y-axis represents log 10 (*p* values). (**e**) Barplot of the DEPs after filtration by adjusted *p* value. Up-regulated DEPs are displayed in red, while down-regulated DEPs are displayed in green.

To understand the impact of ZIC4 over-expression on the protein level, we used SILAC-based quantitative proteomics and compared control CCHE-45 cells to those transiently transfected with ZIC4. Mock control cells were also compared to control cells to account for the effect of the transfection process (Fig. 3a). Upon exclusion of common proteins from the mock/control comparison, MaxQuant search revealed a total of 170 proteins were exclusively detected in ZIC4-transfected cells and not the control cells while 9 proteins were exclusively detected in the control cells (present or absent proteins) (Table S5). Subcellular localization analysis displayed that most of the solely expressed proteins after ZIC4 over-expression were mainly located at the nucleus, especially at the nucleolus and nuclear speckles, as well as endoplasmic reticulum and proteasome (Fig. 3b, and Table S5). Using GO enrichment analysis, we showed that altered proteins clustered in functionally distinct biological processes, including response to endoplasmic reticulum (ER) stress, oxidation–reduction, nucleocytoplasmic transport, and protein transport (Fig. 3c, Table S5). Further, a total of 531 proteins were subjected to differential analysis (Fig. 3d). After the exclusion of common proteins in mock/control comparison and filtering adjusted p-values at cutoff 0.05, a total of 32 differentially expressed proteins (DEPs) were identified (Fig. 3e). Among the top up-regulated proteins was RLIM (E3 ubiquitin-protein ligase RLIM) (fold change = 3.9). In contrast, among the top down-regulated proteins were C1orf109 (Ribosome biogenesis protein C1orf109) (fold change = − 5.08), GLUD1 (Glutamate dehydrogenase 1) (fold change = − 3.92), TRAP1 (Tumor necrosis factor type 1 receptor-associated protein) (fold change = − 2.57) (Table S5).

**Fig. 4:**
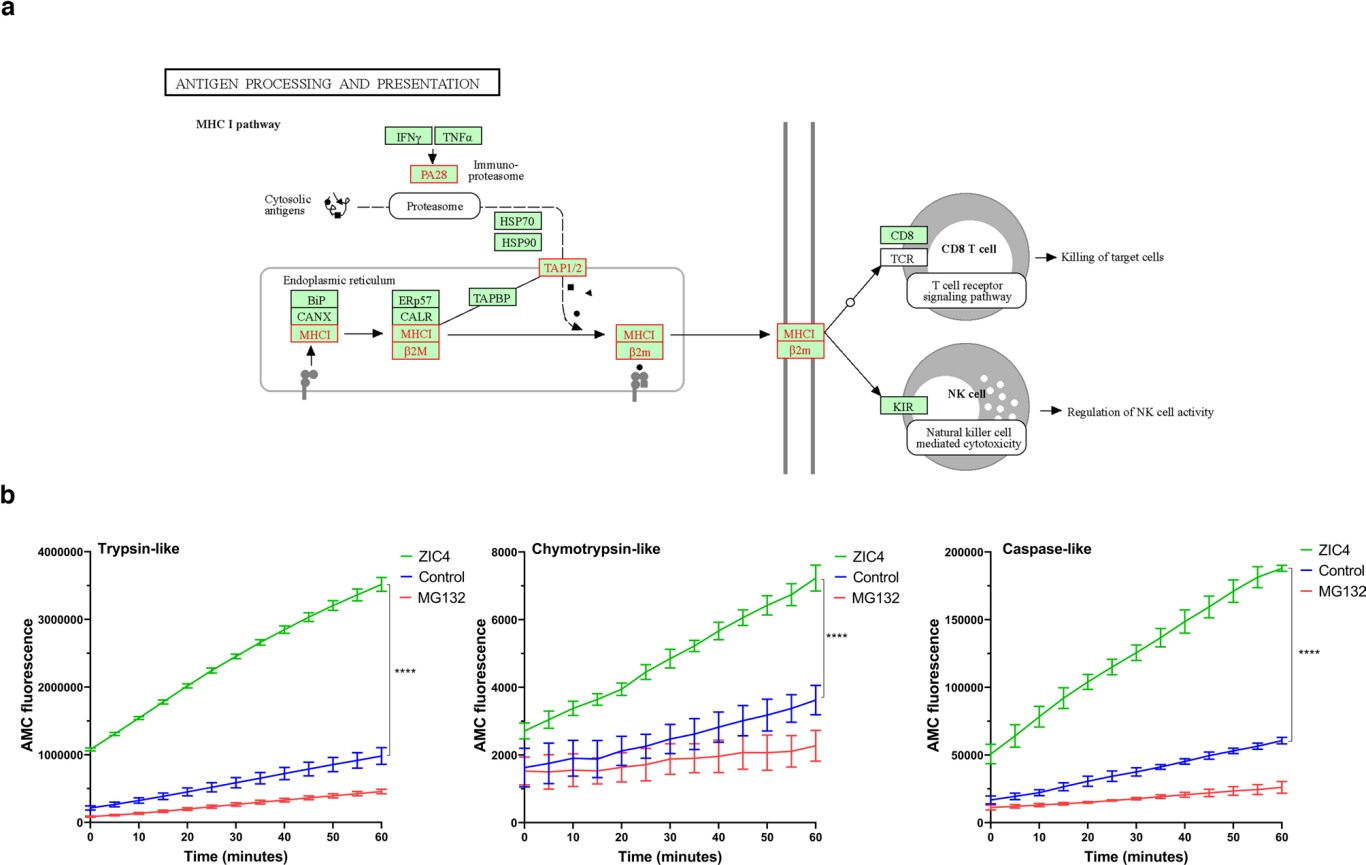
ZIC4 activated proteasome activity potentially for antigen presentation in CCHE-45. (**a**) KEGG plot of antigen presentation and processing pathway shows the increased expression of immune-proteasome subunits, MHC-I (HLA-B), TAP1/2 transporters, and β2M (highlighted in red)^74^. (**b**) ZIC4 increased the proteasome activity in CCHE-45. Proteasome activity assays were performed on protein lysates generated from mock control (blue), MG132-treated (red), or ZIC4-transfected (green) cells to measure trypsin-like, chymotrypsin-like and caspase-like proteasome activity (n = 3; mean ± SD).

Examining RNA-seq and SILAC data, we observed that several pathways identified at the transcriptome level were also reflected at the proteome level. For example, the downregulation of key metabolic proteins such as GLUD1, which is involved in glutamine metabolism and redox homeostasis, aligns with the transcriptomic data showing the depletion of pathways related to oxidative phosphorylation and metabolic processes (Fig. 3c, Table S4). This indicates that ZIC4 over-expression may disrupt metabolic reprogramming essential for tumor cell proliferation. Additionally, the upregulation of immune response and antigen presentation genes at the mRNA level (Fig. 4a) was consistent with the increased expression of proteasomal subunits at the protein level. The activity of the proteasome was assessed after ZIC4 overexpression, and it increased dramatically compared to the mock control (Fig. 4b). This suggests that ZIC4 overexpression modulates gene expression leading to functional changes in protein activity related to immune processing.

## Discussion

In this work, we wanted to understand the role of *ZIC4* gene in CPTs and whether its regulation on the epigenetic level has any clinical and functional implications. Previous whole-genome methylation profiling of CPTs stratified patients into two molecular groups. The first group was associated with a favorable prognosis and included mostly benign CPPs and ACPPs. The second group had a higher risk of tumor progression and mainly was CPCs and few ACPP and CPP^10,29,30^. In the present study, we focused on the methylation status of *ZIC4* in the cohorts mentioned above and we could show that its methylation content correlated with poor prognosis. This finding suggests that the methylation of *ZIC4* gene could serve as a quick molecular marker for CPT aggressiveness. In addition, it was previously reported that DNA methylation of tumor suppressor genes serves to lock chromatin in a specific repressed state that was originally initiated by histone methylation^31^. Our findings suggest that this mechanism can explain the epigenetic silencing of *ZIC4* in the CPC-driven CCHE-45 cell line, mirroring the observations in hepatocellular carcinoma^27^. Epigenetic alterations can contribute to multiple aspects of cancer initiation and progression^8,32^, enabling cancer cells to maintain their fitness while evading a changing immune microenvironment^9,33^.

Transcriptomic and proteomic profiling of CCHE-45 cells after ectopic expression of *ZIC4* highlighted several pathways that could be involved in malignant transformation. On the transcriptome level, ZIC4 increased the expression of genes involved in nucleic acid sensing pathways such as RIGI-like receptor and interferon (IFN)-signaling. RIG-I is a cytoplasmic pattern recognition receptor known to detect immunostimulatory RNA, for example, viral RNAs in infected cells, and mediate immune defense against pathogens via type 1 IFN response^34^. Recent studies have shown that RIG-I can also detect specific types of aberrant RNA associated with cancer, resulting from alteration in RNA metabolism, mislocalization, or defects in RNA processing^35,36^. Indeed, the activation of the RIG-I pathway in cancer cells was shown to contribute to the antitumor immune response by generating an inflammatory tumor microenvironment^37,38^. Additionally, ZIC4 over-expression increased the expression of *STAT1*, an essential mediator of IFN signaling, a subset of ISGs (*IRF*s, *IFIT*s, *IFITM*, and the *MX*, *OAS* family members), and *XAF1*, a pro-apoptotic tumor suppressor gene^39^. It has previously been shown that the ISGs we observed can inhibit cell proliferation and stimulate cancer cell death^40–42^. XAF1 has been shown to inhibit proliferation and induce apoptosis and ER stress in cancer cells^39,43,44^. The impact of reintroducing ZIC4 on innate immune response in our model system could also be seen with other zinc finger proteins, like BNC2 (human basonuclin 2) and ZNF395 in cancer cells^45,46^. Finally, ZIC4 over-expression increased the expression of key antigen processing and presentation genes such as immune-proteasome subunits (*PSMB8* and *PSMB9*), *HLA-B, TAP1/2* transporters, and *β2M* microglobulin, which is a component of MHC-I^47^. It has been previously shown that the downregulation of HLA-1 promotes immune evasion in prostate cancer^48^. Therefore, we can hypothesize that our CPC model system invokes similar immune evasion mechanisms through epigenetic modifications.

On the proteome level, ZIC4 over-expression led to the up-regulation of proteins involved in ER and oxidative stress down-regulation of essential metabolism proteins like glutamate dehydrogenase1 protein (GDH1/ GLUD1). GLUD1 can regulate redox homeostasis in cancer cells by metabolic reprogramming in terms of enhancing aerobic glycolysis and protein translation, which are vital for tumor proliferation^49,50^. Hence, this implies that glutamine metabolism may support the hyperproliferation state of CCHE-45 cells.

The integration of RNA-seq and SILAC data provided a prospective understanding of the molecular mechanisms underlying ZIC4's tumor suppressor role in CPTs. The upregulation of immune response genes and proteins, and those involved in antigen processing and presentation, suggests that ZIC4 over-expression enhances the immunogenicity of CPC cells. This is further supported by the increased proteasome activity, which is crucial in generating peptides for MHC class I presentation. It is well known that the proteasome, specifically the immuno-proteasome, is responsible for the degradation of proteins into smaller peptides, which are then transported into the ER for loading onto MHC molecules^51^. In addition, the observed downregulation of essential metabolic proteins such as GLUD1, along with the depletion of oxidative phosphorylation pathways on the transcriptome level, indicates that ZIC4 over-expression disrupts the metabolic adaptations commonly seen in cancer cells by impairing glutamine metabolism and redox homeostasis. Hence, ZIC4 may hinder the ability of CPC cells to sustain rapid proliferation and survival.

It is important to note that the transcriptome profile we observed after ZIC4 over-expression was not entirely reflected on the proteome level, and that is not surprising considering that the proteome offers a picture of processes that are not transcriptionally regulated^52,53^. Alternatively, the discrepancy can be attributed to the varying sensitivity and detection limits of the assays used, which arise from differences in experimental techniques and bioinformatics approaches^54^. For example, up-regulation of ISGs was detected in the transcriptome and not in the proteome, and a downstream impact of this could be seen in our proteomic analysis as signs of ER stress^55^.

In conclusion, our findings support a picture whereby epigenetic silencing of key transcription factors such as *ZIC4* gene can drive carcinogenesis in CPTs. *ZIC4* methylation was correlated to cancer aggressiveness and could, therefore, be used as a prognostic molecular marker. Using an in vitro model system to understand the underlying mechanisms and pathways involved, we could show that epigenetic silencing of *ZIC4* is essential for establishing key cancer hallmarks such as increased proliferation, migration, and potential immune evasion. Reintroducing ZIC4 in this context could be a novel way to target CPTs, and this can be done, for example, with novel RNA therapeutics^56^.

## Methods

### Bioinformatics and survival analysis

We utilized genome-wide methylation data of pediatric CPTs from two independent datasets, GSE61044^30^ and GSE156090^10^, consisting of 31 and 42 samples, respectively. The beta-values (β-values) and the genomic coordinates for the corresponding probes (CpG site) representing ZIC4 in each cohort were extracted (Table S1). Methylation status analysis and visualization were performed using the Methylation plotter tool^57^. A methylation profile plot summarizing the methylation status of *ZIC4* CpG sites in different CPT subtypes and a dendrogram that classifies the data by unsupervised clustering were generated. A gray color gradient represents methylation values ranging from 0 (unmethylated) to 1 (fully methylated). Survival analysis of the GSE156090 cohort was performed using Kaplan–Meier analysis in SPSS software. Downstream targets of ZIC4 were identified from transcription factor databases, including TF2DNA^58^ and TFBSDB^59^. Additionally, the EZH2 binding site on *ZIC4* was identified by MotEvo, a Bayesian probabilistic method for inferring regulatory sites and motifs like transcription factor binding sites and enhancers on multiple alignments of DNA sequences^60^.

### Cell culture, drug treatment, and plasmids

CCHE-45 cells^61^ were cultured in Roswell Park Memorial Institute-1640 (RPMI 1640) media (Gibco) supplemented with 10% fetal bovine serum (FBS) (Gibco) and 1% PEN-STREP (Gibco) at 37 °C in a humidified 5% CO2 incubator. Both 3-Deazaneplanocin A (DZNep) (APExBIO, A8182) and 5-aza-2′-deoxycytidine (5-Aza-Dc) (Sigma-Aldrich, 189825-25MG) were dissolved in DMSO. For the 5-Aza-dC treatment, cells were kept in media supplemented with 5-aza-dC at a final concentration of 10uM. The media was replaced daily for 72 h. Cells treated with 10 μM DZNep were added to the culture medium for 72 h. CCHE-45 cells were transfected using the GFP-tagged-ZIC4 expression vector (Origene) and the GFP vector (Clontech) with Linear Polyethylenimine Hydrochloride PEI MAX® (Polyscience). The cells were pre-seeded one day before transfection to achieve a 70%–80% confluency. Further, we performed the MTT assay as previously described^62,63^. Cells were plated at a density of 1 × 10^4^ cells per well in 96-well plates. The experimental conditions included untransfected control cells, cells transfected with an empty GFP vector (mock control), and cells transfected with the ZIC4 vector. The transfected cells were then incubated for 48 h in a 5% CO2 environment at 37 °C. Each condition was performed in triplicate.

### RNA extraction and quantitative reverse transcription-polymerase chain reaction (Qrt-PCR)

Total RNA was extracted from cultured cells using GeneJet RNA purification kit (Thermo Scientific, K0731) according to the manufacturer’s instructions. RNA quantity and quality were determined by a NanoDrop ND-1000 Spectrophotometer. The cDNA was synthesized from total RNA using the RevertAid first-strand cDNA synthesis kit as described by the manufacturer (Thermo Scientific, K1621). Real-time PCR was carried out on CFX96 Touch Real-Time PCR Detection System (Bio-Rad) using HERA SYBR Green qPCR kit (Willowfort, WF10308001) as described by the manufacturer. The relative expression levels were evaluated by using the 2^−ΔΔCt^ method. Primers for each gene were listed in (Table S2). All results were expressed as the mean ± standard deviation (S.D.) of three independent experiments.

### Bisulfite sequencing

To identify the methylation status of *ZIC4*, DNA-bisulfite conversion followed by TA-cloning assay and sequencing were performed on CCHE-45 cells. First, primers for bisulfite sequencing were designed to cover all CpG islands of ZIC4 using MethPrimer (Table S2). Genomic DNA was extracted from the cell line and subjected to bisulfite- treatment using Zymo DNA modification kit (Zymo Research, USA). The bisulfite-converted DNA was then used for TA-cloning using a TOPO TA cloning kit (Invitrogen, 45-1641). Subsequently, four colonies were selected per CpG island, and plasmid DNA was isolated for Sanger sequencing using BigDye terminator v3.1 cycle sequencing kit (Applied Biosystems, 4336917). Visualization and analysis of DNA methylation results from bisulfite sequencing was performed using QUMA (quantification tool for methylation analysis)^64^.

### Western blot analysis

Cultured cells were collected and lysed using urea extraction buffer (8 M urea, 500 mM Tris–HCl (pH = 8.5) with a protease inhibitor cocktail (Thermo Scientific). After the cells were lysed, supernatants were collected, and total protein concentrations were measured by Bradford assay (Thermo Scientific, 23,200). A total of 50 ug of proteins were resolved by SDS–PAGE and transferred to a PVDF membrane (Thermo Scientific). The membranes were then blocked by 5% non-fat dry milk (Cell signaling) and incubated with appropriate primary antibodies at 4 °C overnight. Subsequently, they were incubated by HRP-conjugated secondary antibody. Signal detection was performed using Pierce ECL plus W.B. substrate (Thermo Scientific, 32132) and scanned using the ChemiDoc MP Imaging System (Bio-Rad). Antibodies used in the study include anti-H3K27me3(diluted at 1:1000) (Cell Signaling, 9733), anti-EZH2(diluted at 1:2000) (Thermo Scientific, #MA5-18108), anti-ZIC4 (diluted at 1:100) (Santa Cruz, sc-101202), anti-GAPDH (diluted at 1:1000) (Abcam, ab179467), goat anti-rabbit IgG H&L(HRP) (diluted at 1:1500) (Abcam, ab6721) and rabbit anti-mouse IgG H&L (HRP) (diluted at 1:1500) (Abcam, ab6728).

### Immunofluorescence and imaging

Control and transfected cells were seeded in a 96-well imaging plate pre-coated with collagen for 1 h. Afterward, cells were fixed with 4% paraformaldehyde for 15 min and permeabilized using 0.3% Triton X-100 in phosphate-buffered saline (PBS) for 15 min. Next, cells were treated with blocking buffer (0.3% Triton X-100 and 5% FBS in PBS), which was added to cells for 1 h at room temperature. Fixed cells were then incubated with ZIC4 primary antibody (1: 50 dilution) (Santa Cruz, sc-101202) in antibody-dilution buffer (0.3% Triton-X and 1% BSA) overnight at 4˚C then washed three times with 1 × phosphate buffer saline (PBS). Cells were then incubated with secondary antibody (Invitrogen, A21430) for 1 h in the dark at room temperature, followed by incubation with DAPI (Invitrogen, D1306) for nuclear staining. Imaging was performed using a ZEISS LSM 980 with an Airyscan 2 confocal microscope at 63X magnification with a Plan-Apochromat 63x/1.4 Oil DIC.

### Scratch wound healing assay

The cells were cultured at a density of 1 × 10^6^ cells/well in a 6-well plate for the scratch wound healing assay and incubated overnight at 37 °C and 5% CO_2_. The next day, a scratch wound was carefully created at the center of the confluent monolayer cells using a 10-μL sterile tip. After removing any cellular debris by thorough washing with PBS, the cells were transfected in fresh media. A confocal microscope (LSM 980) equipped with a T-PMT detector was used to observe the cell migration and to image the scratches, followed by further incubation at 37 ºC and 5% CO_2_. The effect of the transient transfection of ZIC4 on cell migration in the scratch area, compared with the control cells, was observed at 0, 24, and 48 h. To quantitatively assess scratch closure, the wound area at times 0, 24, and 48 h was recorded, and the differences were calculated using the Zen Blue 3.3 software^65^. The wound closure rate was calculated according to the following equation:

$$Wound\;closure\;{\text{rate }}\left( {{\mu m}^{2} {\text{/h}}} \right){ } = \frac{{{\text{W}}0{ } - {\text{ Wt}}}}{{\Delta {\text{T}}}}$$

where W0, wound area at 0 h (µm^2^); Wt, wound area at ∆h (24 or 48 h) (µm^2^), ∆T, duration of wound measured (h)^66^. Data are presented as mean ± S.D. Three replicates were included in the analysis, and an unpaired Student's t-test was performed. Significance was considered at *p* < 0.05.

### RNA sequencing and data analysis

The quantity and quality of the RNA samples from mock control (n = 3) and ZIC4-transfected cells (n = 3) were assessed using the following methods. Preliminary quality control was performed on 1% agarose gel electrophoresis to test RNA degradation and potential contamination. Sample purity, quantitation, and RNA integrity were further evaluated using the Bioanalyzer 2100 (Agilent Technologies, USA). For library preparation, the mRNA was isolated with magnetic beads of oligos d(T)25 for polyA-tailed mRNA enrichment. Subsequently, mRNA was randomly fragmented, and cDNA synthesis was done using random hexamers and the reverse transcriptase enzyme. Once the synthesis of the first chain was finished, Illumina buffer (non-directional library preparation), dNTPs, RNase H, and polymerase I from *E. coli* were added, and the second chain was obtained by Nick translation. The resulting products were subjected to purification, end-repair, A-tailing, and adapter ligation. Fragments were then enriched by PCR, where indexed P5 and P7 primers were introduced, and final products were purified. The library was assessed with Qubit 2.0 and real-time PCR for quantification and the Agilent 2100 bioanalyzer for size distribution detection. Pooled and quantified libraries were sequenced on the Illumina Novaseq 6000 platform with paired-end strategy 150 bp (PE150). For data analysis, the Kallisto (version 0.46.1)^67^ index function was used to generate a reference index from ENSEMBL GRCh38.p14 human genome (version 111.38). The reference index was then used to generate the abundance transcript matrix from the fastq files using the kallisto quant function. Gene-level summaries and differential expression analysis were performed using sleuth with Wald test^68^. Differentially expressed genes (DEGs) were identified at adjusted *p* values less than 0.05 and fold change between (1.5 and − 1.5).

### SILAC and mass spectrometry (LC–MS/MS)

Stable isotope labeling of amino acid in cell culture (SILAC)-coupled quantitative mass spectrometry proteomics analysis was performed to identify and quantify proteins affected by ZIC4 over-expression. CCHE-45 cells were cultured under light media conditions for those transfected with ZIC4 or mock control, while untreated control CCHE-45 cells were cultured in heavy media supplemented with 13C6 L-Arginine isotope (Cambridge Isotope Laboratories). Lysate protein quantification was performed using Pierce™ BCA Protein Assay Kit (Thermofisher Scientific), and light and heavy protein lysates were mixed in equal amounts (1:1) and subjected to LC–MS/MS treatment and analysis.

For LC–MS/MS, standard in-solution digestion with trypsin was performed using 30ug of each sample, followed by desalting the sample using Pierce™ C18 Spin Tips (Thermofisher Scientific). MaxQuant software (version 1.6.17)^69^ was used to analyze raw files to identify and quantify peptides. Peak lists were searched against the human UniProt database. Heavy label feature was set to Arg6, and modifications of methionine oxidation and acetylation of the protein N terminus were selected. A maximum of two missed cleavages were allowed in the search, and the false discovery rate (FDR) was set to 1% at both the peptide and protein levels. The 'Match between runs' option was chosen, and unique + razor peptides were selected for quantification while all other settings were set to the default. Present or absent proteins were identified according to their corresponding intensity per condition. Those with detected intensity at the light condition and zero intensity at the heavy condition will be considered exclusively detected in light, and vice versa. Differential expression analysis was performed using ProteoSign V2^70^. Contaminants were removed, and differentially expressed proteins were detected at adjusted *p* value cutoff < 0.05 and log2 fold-change between (1.5 and − 1.5).

### Proteasome activity assay

Proteasome activity in protein lysates of CCHE-45 under various conditions; proteasome inhibitor MG132-treated (Cell signaling, 2194S), mock control, and ZIC4-transfected were assessed according to the protocol described by Vilchez et al., 2012^71^. Specifically, cells were cultured and subsequently collected by centrifugation. The cells were immediately re-suspended in a proteasome activity buffer consisting of 50 mM Tris–HCl (pH 7.5), 250 mM sucrose, 5 mM MgCl_2_, 0.5 mM EDTA, and 1 mM dithiothreitol. The lysates were obtained by passing the cells through a 27-gauge needle ten times, followed by centrifugation at 15,000×*g* for 10 min at 4 °C to remove debris. The protein concentration was determined using the Pierce™ Bradford protein assay (Thermofisher Scientific, 23238). Subsequently, 15 μg of protein was loaded into the wells of a flat bottom 96-well plate for the assay. Proteins were loaded equally in each well, and 2 mM adenosine triphosphate and 0.37 mM of the respective proteasome substrates (caspase—Z-Leu-Leu-Glu-AMC, chymotrypsin—Suc-Leu-Leu-Val-Tyr-AMC, trypsin—Boc-Leu-Arg-Arg-AMC) were added. The wells were then diluted with proteasome activity buffer to a final volume of 100 μL per well. The plate was immediately placed in a plate reader set to excite at 380 nm and collect at 460 nm, with data recorded every 5 min for 1 h at 37 °C. Three 96-well reactions were averaged for each technical replicate and repeated on three separate days. For each substrate, a reaction with no protein was subtracted from each experimental condition to account for background reading. Normality testing was carried out as described in^72^. Statistical comparisons were made using the Student's t-test for unpaired samples in each condition.

### Network and enrichment analysis

Gene Set Enrichment Analysis (GSEA, version 4.0.3) was conducted using the gene lists and values from Kallisto output^73^. The enrichment analysis was performed using the Kyoto Encyclopedia of Genes and Genomes (KEGG) gene set^74^. The leading edge analysis was also conducted on the top up-regulated genes, allowing the determination of genes that made the most significant contribution to the enrichment signal of a specific gene set's leading edge or core enrichment. WebGestalt (WEB-based Gene SeT Analysis Toolkit)^75^ was used to perform over-representation enrichment analysis of the differentially expressed proteins (DEPs). Gene ontology (Biological Process) was identified based on (Benjamini- Hochberg FDR < 0.05) with the removal of redundant terms. NetworkAnalyst^76^ was employed to generate a protein–protein interaction network using the STRING database.

## Statistical analysis

Statistical analyses were performed using GraphPad Prism 8 software. All results are presented as mean ± standard deviation from at least three independent experiments. Student t-tests were performed to analyze the differences between the two groups. Differences were considered statistically significant when *P* values < 0.05. In all figures, statistical significances were denoted as *P < 0.05, **P < 0.05, and ***P < 0.005.

## Supplementary Information

Supplementary Information 1.

Supplementary Information 2.

Supplementary Information 3.

Supplementary Information 4.

Supplementary Information 5.

Supplementary Information 6.

## Data Availability

The proteomics data generated and analyzed during the current study are available in the (PRIDE) repository under the accession ID PXD043748. RNA-sequencing data were deposited at EMBL Biostudies with accession ID S-BSST1366.
