# Supplementary material for: Epigenetic silencing of ZIC4 unveils a potential tumor suppressor role in pediatric choroid plexus carcinoma
Source: Sci Rep. 2024 Sep 12;14:21293. doi: 10.1038/s41598-024-71188-7 (PMC11393135; doi:10.1038/s41598-024-71188-7)
Supplement: Supplementary file 1 — Supplementary Information 1. [file 41598_2024_71188_MOESM1_ESM.docx]

**
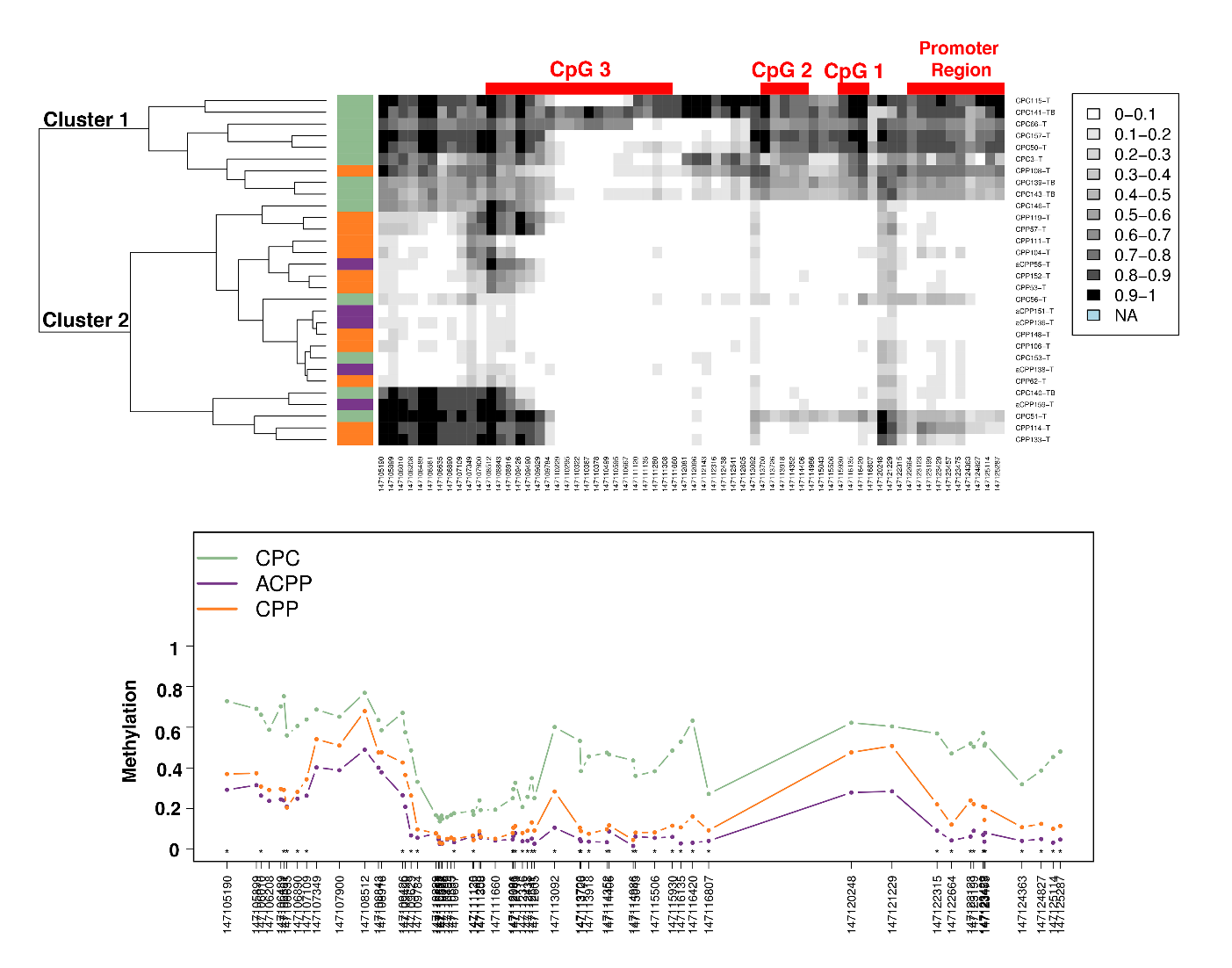
**

**Figure S1. *ZIC4* methylation in status GSE61044 cohort**. β-values of *ZIC4* methylation probes segregated CPTs into 2 clusters: high-methylation “ cluster 1" and low-methylation “cluster 2",as shown in the dendogram of GSE61044 cohort. The positions representing CpG islands and promoter regions are highlighted in red. In the methylation plot, each line represents the methylation mean for each position for each group of samples. Asterisks indicate a statistical significance as calculated by the Kruskal-Wallis test. The coloring of the samples was according to the pathology (CPC=green, ACPP=purple and CPP=orange).

**
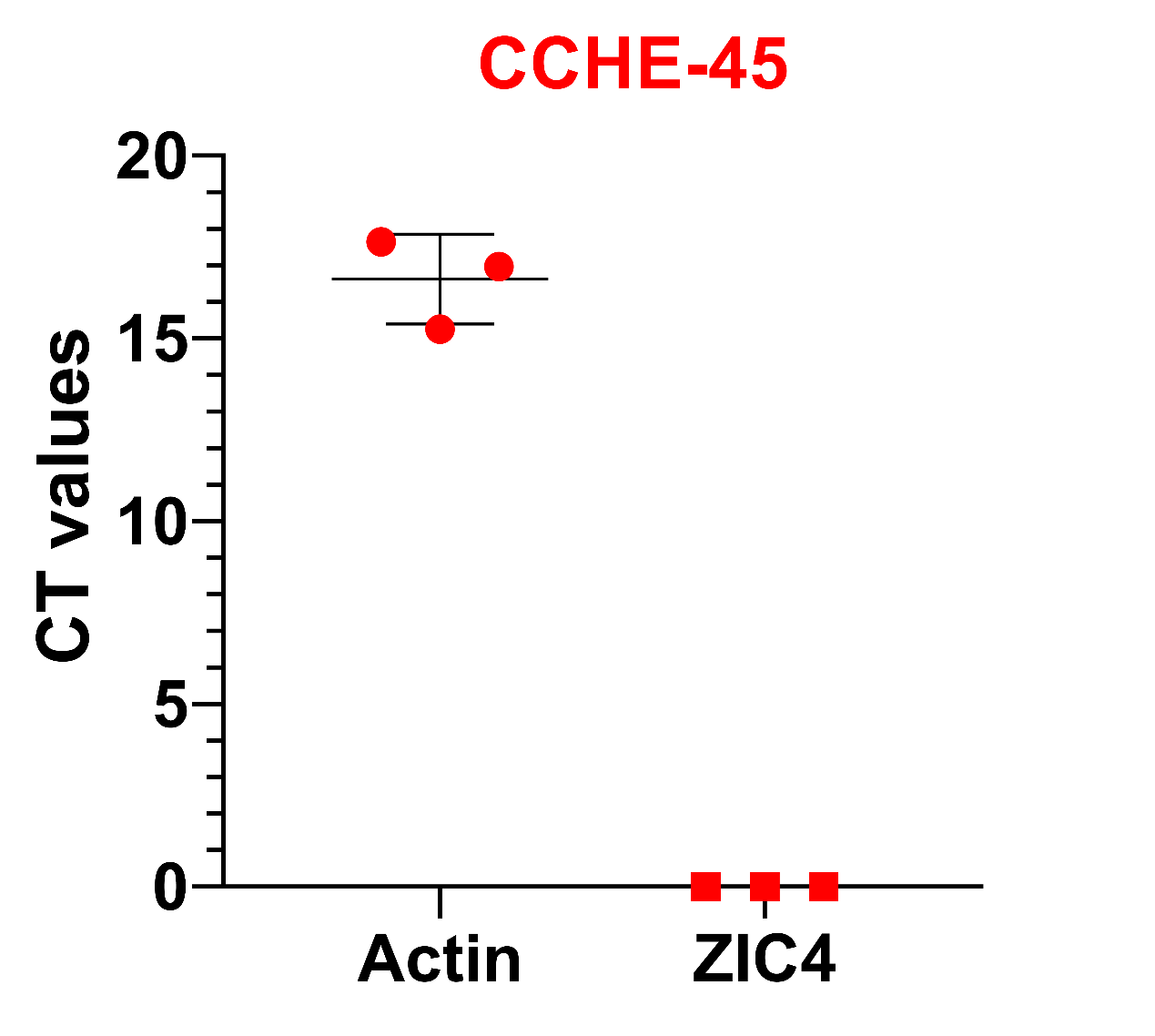
**

**Figure S2. *ZIC4* expression is absent in CCHE-45.** mRNA expression of *ZIC4* was determined using RT-qPCR in CCHE-45 cell line (n=3). Actin was used as an internal control.

**
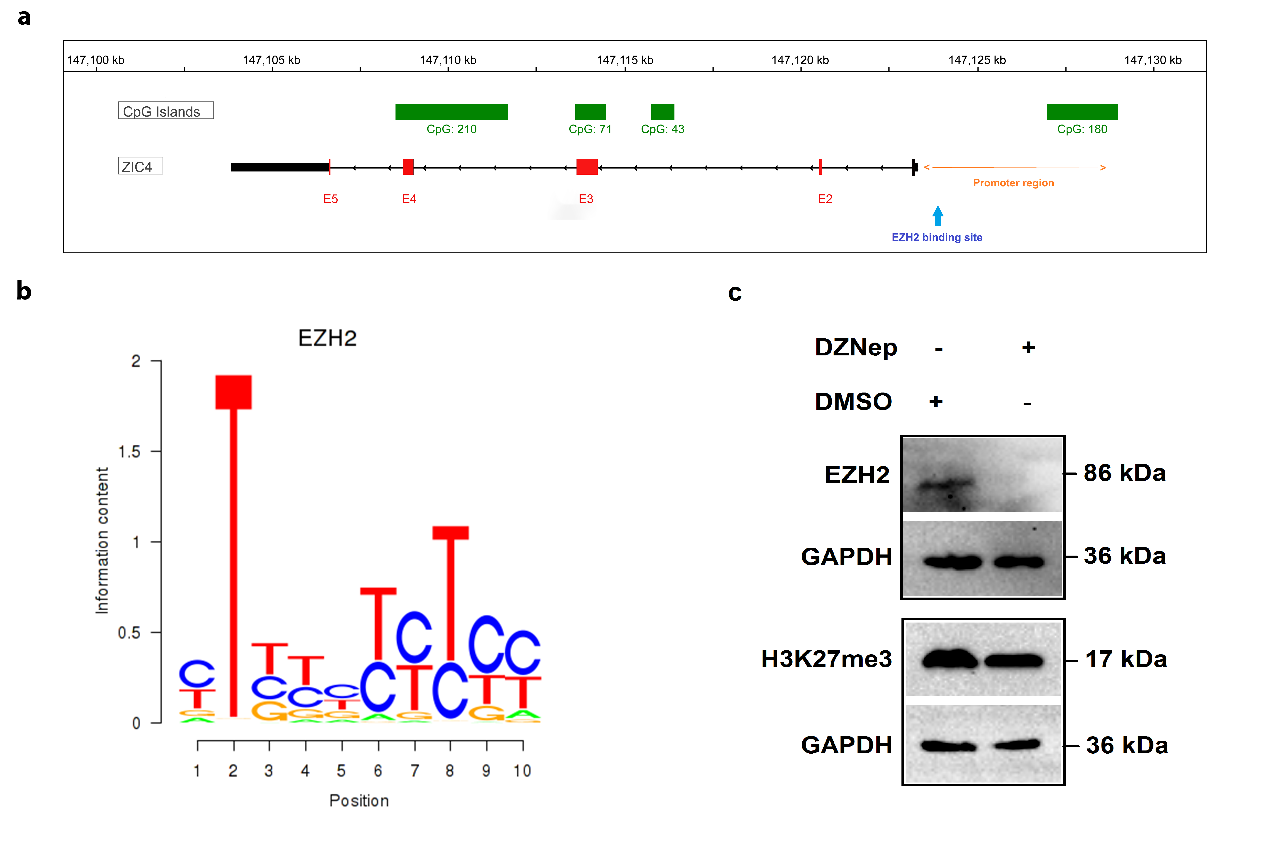
**

**Figure S3. Epigenetic regulation of *ZIC4*.** (a) A schematic representation of *ZIC4* gene showing the methylation CpG islands, coding exons, and promoter region. (b) EZH2 binding site on *ZIC4*, inferred computationally by MotEvo. (c) Western blot analysis of H3K27me3, EZH2 and GAPDH in CCHE-45 with DMSO (control) or DZNep (10 μM) treatment for 72 h.

**
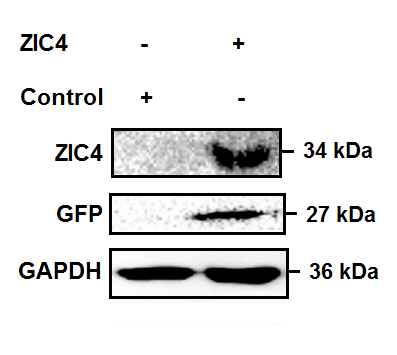
**

**Figure S4. Western blot analysis after ZIC4 over-expression in CCHE-45 cells.** Both ZIC4 and GFP antibodies to detect the expression of ZIC4 and the GFP tag in ZIC4-transfected vs control cells.

**
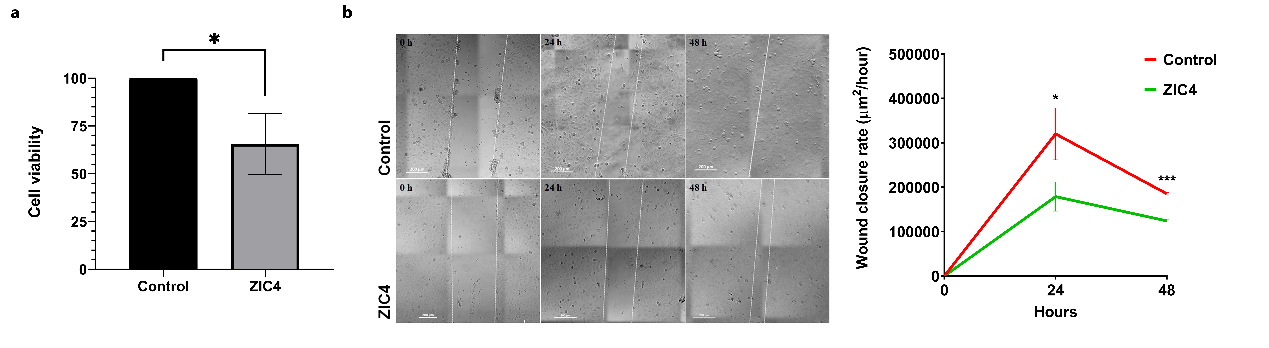
**

**Figure S5. Re-expression of *ZIC4* affects cancer properties in CCHE-45.** (a) MTT assay was performed to detect cell viability of cells in control cells and ZIC4-transfected cells (n=3). (b) Scratch wound healing assay was performed to detect cell migration in control cells and ZIC4-transfected cells (n=3). Representative results of wound-healing assay and wound closure rate at different time points. *P<0.05, ***P<0.005.

**
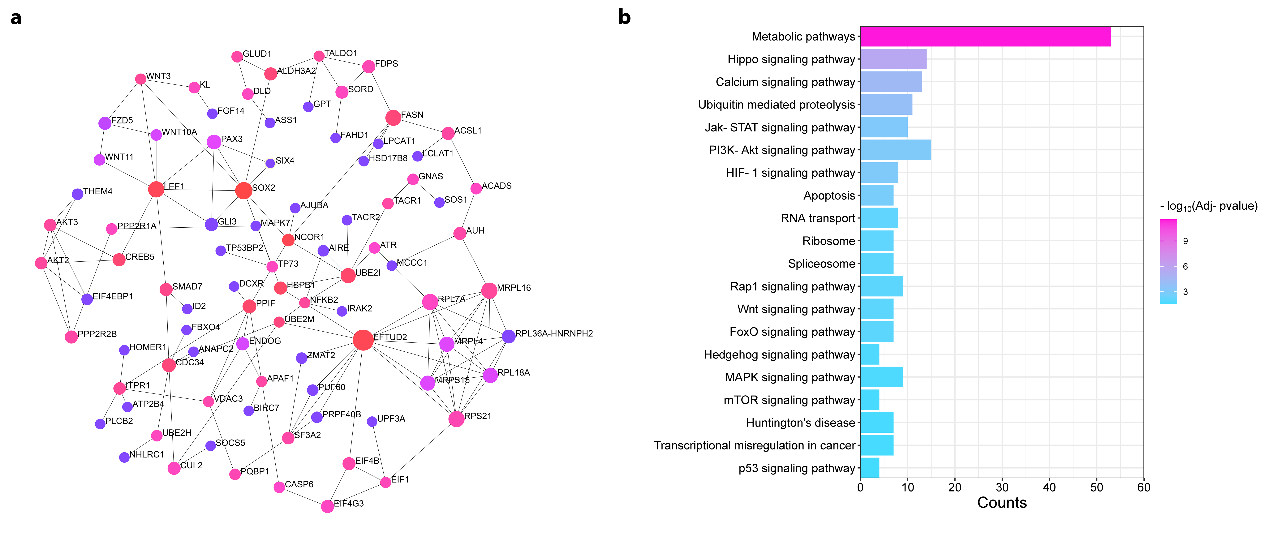
**

**Figure S6**. **Downstream targets of *ZIC4* are involved in various cancer-related pathways**. (a) String protein-protein interaction (PPI) network of interacting downstream targets of ZIC4 by NetworkAnalyst. (b) KEGG pathways enriched by the interacting downstream targets of ZIC4, Counts represents the number of genes from the geneset per pathway. Color gradient of the bars is according to the –log of Adjusted-pvalue.


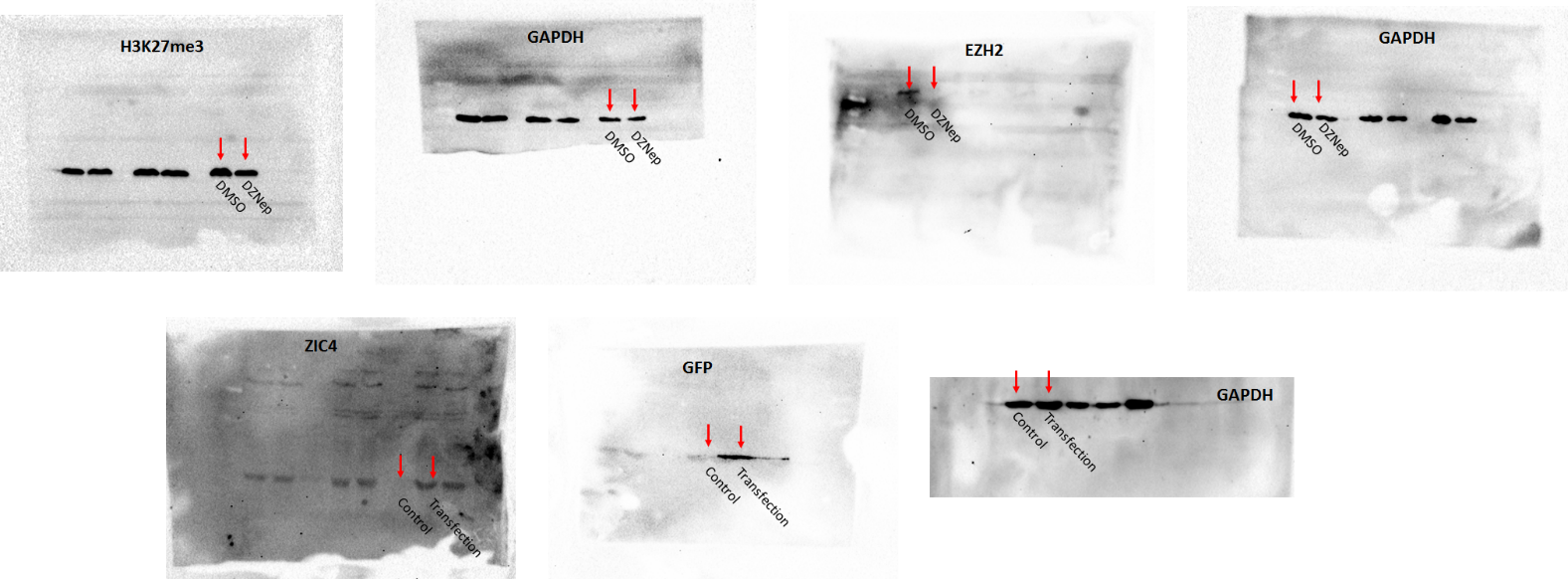


**Figure S7**. Original western blots for EZH2/GAPDH, H3K27me3/GAPDH, and ZIC4/GFP/GAPDH, respectively. For GAPDH blots, the membrane was cut prior to blocking and hybridization as the rest of the membrane was used for other purpose.
